# Treatment of Uterine Fibroid–Related Heavy Menstrual Bleeding: Variations in Clinical Practice at Four Hospitals in the Netherlands

**DOI:** 10.1155/ogi/2889686

**Published:** 2026-02-25

**Authors:** Elisabeth R. Knorren, Daniëlle P. C. Huijs, Ingrid M. Nijholt, Jeroen R. Dijkstra, F. Paul H. L. J. Dijkhuizen, Jan Willem van der Steeg, Tycho van der Meer, Martijn F. Boomsma, Maarten D. H. Vink, Peggy M. A. J. Geomini, Marlies Y. Bongers, Jaklien C. Leemans

**Affiliations:** ^1^ Department of Radiology, Isala, Zwolle, the Netherlands, isala.nl; ^2^ Department of Obstetrics and Gynecology, Máxima Medical Centre, Veldhoven, the Netherlands, mmc.nl; ^3^ GROW School for Oncology and Reproduction, Maastricht University, Maastricht, the Netherlands, maastrichtuniversity.nl; ^4^ Department of Obstetrics and Gynecology, Isala, Zwolle, the Netherlands, isala.nl; ^5^ Department of Obstetrics and Gynecology, Rijnstate Hospital, Arnhem, the Netherlands, rijnstate.nl; ^6^ Department of Obstetrics and Gynecology, Jeroen Bosch Hospital, s-Hertogenbosch, the Netherlands, jbz.nl; ^7^ Department of Obstetrics and Gynecology, Noordwest Ziekenhuisgroep, Alkmaar, the Netherlands, nwz.nl; ^8^ Department of Obstetrics and Gynecology, Meander Medical Center, Amersfoort, the Netherlands, meandermc.nl

**Keywords:** clinical practice patterns, clinical practice variation, heavy menstrual bleeding, leiomyoma, therapeutics

## Abstract

**Introduction:**

Uterine fibroids are the most common anatomical cause of heavy menstrual bleeding. To which extent clinical practice variation is present in the treatment of fibroid‐related heavy menstrual bleeding at the hospital level remains unclear. The aim of this study was to identify and evaluate the clinical practice variation in the treatment of fibroid‐related heavy menstrual bleeding.

**Material and methods:**

In this multicenter, retrospective database study, pseudonymized real‐world data were collected from electronic health records using a natural language processing and text‐mining data collection tool. Women ≤ 55 years, who presented as new patients at the gynecology outpatient clinic in 2019 with heavy menstrual bleeding and fibroids, were selected. Data were extracted from the first appointment in 2019 throughout December 2022. The primary outcome was the number of treatments initiated. Secondary outcomes were the type of treatments initiated, treatments initiated prior to hysterectomy, and time to hysterectomy.

**Results:**

From four hospitals, 623 women were included. The median age was 46 (range: 23–55) years. Overall, a median of one treatment (range: 1–4) was initiated, which significantly differed between hospitals (*p* < 0.01). Pharmacological treatment was initiated most frequently, which differed significantly among hospitals (392/623 [62.9%], range: 49.1%–70.5%, *p* = 0.02). Minimally invasive therapies were initiated in 51.2% (319/623, range: 40.6%–58.9%, *p* < 0.01). Only 30/319 patients (9.4%) received a minimally invasive uterus‐sparing treatment. Hysterectomy was performed in 123/319 patients (38.6%), with Hospital 1 being an outlier as 52.3% underwent hysterectomy, compared to 23.9%–36.4% in the other hospitals (*p* < 0.01).

**Conclusions:**

Clinical practice variation is present in the treatment of heavy menstrual bleeding in Dutch women with fibroids. Organizational factors could partially explain clinical practice variation. Sharing data can aid in identifying, explaining, and acting on (un)warranted practice variation between healthcare clinics. While clinical practice variation remains inevitable, unwarranted practice variation should be limited by ameliorating guideline adherence, educational interventions, and patient counseling, to improve the quality, efficiency, and equity of care.

**Key Message**
 A certain amount of clinical practice variation remains inevitable in the treatment of fibroid‐related heavy menstrual bleeding. Unwarranted variation should be limited by ameliorating guideline adherence and educational interventions, encouraging intercollegiate consultation, and improving patient counseling, to improve the quality, efficiency, and equity of care.

## 1. Introduction

Heavy menstrual bleeding (HMB) is defined as the presence of > 80 mL blood loss during a period or as excessive menstrual blood loss affecting the menstruating person’s physical, social, emotional, and/or material quality of life regardless of the amount of blood loss [1]. The exact prevalence of HMB is unknown but is estimated to occur in 20%–51% of menstruating women, making it one of the most common reasons for gynecological consultation [2, 3]. The most common structural cause for HMB is uterine fibroids [3]. Uterine fibroids occur in 5%–70% of reproductive women, depending on the study population and diagnostic measures [4, 5]. Ethnicity has been identified as the most significant risk factor in the development of uterine fibroids. Black women are two to three times more likely to develop uterine fibroids compared to White women. Also, East and South Asian and Hispanic women are more likely to develop fibroids than White women [6]. In Eastern European countries, the self‐reported prevalence of uterine fibroids is 11.7%–23.6% [7]. Approximately 50% of women with uterine fibroids are symptomatic, of which 25% seek treatment [1, 4]. Patients often present with a combination of symptoms. HMB is the most common symptom, followed by mechanical complaints, pain, and sub/infertility, respectively, estimated to occur in 40%, 30%, 15%, and 2%–3% of women with uterine fibroids [8, 9].

Several treatment options are available, ranging from conservative (non)hormonal pharmacological treatment to surgical interventions, such as hysterectomy. Whether a treatment option is appropriate is influenced by location, symptoms, size and number of fibroids, and patient’s preferences (e.g., fertility/uterus preservation). Hence, a personalized treatment plan is required to address the variability in these characteristics. International guidelines recommend a stepwise treatment model, starting with pharmacological treatment, when possible, followed by minimally invasive treatment options when indicated, and finally surgical treatment [5, 10, 11].

Clinical practice variation (CPV) refers to the variation in the provision of healthcare to patients with similar health problems. CPV may be warranted, for example, by differences in patient populations and their preferences, or unwarranted when it cannot be explained by these differences, potentially resulting from a lack of consensus or necessary facilities, poor guideline adherence, or reluctance of the attending physician to implement innovations [12]. Identification of (un)warranted CPV facilitates the improvement of quality, efficiency, and equity of care [13, 14].

Regional CPV has been evaluated for the treatment of fibroids with therapeutic hysteroscopy, endometrial ablation, uterine artery embolization (UAE), myomectomy, and hysterectomy [15]. However, the rate of CPV in the treatment of HMB in women with fibroids has not been specifically investigated, particularly at a lower aggregation level, such as hospital level. By understanding where and why treatment decisions differ, healthcare systems can better align practices with evidence‐based guidelines, reduce unnecessary interventions, and ensure that patients receive consistent and appropriate care regardless of where they are treated. Therefore, the aim of this retrospective database study was to identify and evaluate CPV for the treatment of HMB caused by fibroids in the Netherlands.

## 2. Materials and Methods

This multicenter, retrospective database study was designed to collect pseudonymized real‐world data from electronic health records (EHR).

The Medical Ethics Committee of the initiating hospital evaluated the study protocol (MEC Number: N22.037) and deemed it not applicable to the Medical Research Involving Human Subjects Act (WMO). Local feasibility approval was granted by the local feasibility research committees of participating centers. Following the European General Data Protection Regulation, informed consent was not required.

### 2.1. Dutch Healthcare System

Dutch citizens are obliged to have healthcare insurance that covers primary and hospital care. To facilitate reimbursement by the health insurance company, a system of diagnosis treatment combination (DTC) is used. DTCs are diagnosis specific and cover the mean healthcare costs for that diagnosis, including diagnostic assessments, pharmaceuticals, and interventions. These interventions have DTC‐related healthcare activity codes (HCAC). The use of a DTC system reduces financial incentives to initiate more or more expensive treatments, as reimbursement is derived from the most expensive care product in the DTC system within 3 months after opening the DTC [16].

### 2.2. Clinical Data Collector

The database was created using the Clinical Data Collector (CDC) module of CTcue (CTcue B.V., Amsterdam, the Netherlands). The CDC is a natural language processing and text‐mining–based tool, which can pseudonymously collect structured (such as appointments, diagnosis and procedure codes, medication, and time logs) and unstructured (such as HMB, body mass index [BMI], and International Federation of Gynecology and Obstetrics [FIGO] classification) data from EHR [17]. Five teaching hospitals in the Netherlands participated in this study. Data were extracted using the CDC linked to the local EHR.

### 2.3. Patient Selection

Patients were included if (1) they visited the gynecology outpatient clinic as a new patient in 2019; (2) DTC G11 (cycle disorders) or G15 (uterine fibroids) was registered within one month after the date of the first consultation; (3) age was ≤ 55 years at registration of the DTC; (4) the diagnosis code or term “heavy menstrual bleeding” (including synonyms) was registered in the EHR in 2019; and (5) the diagnosis code or term “uterine fibroids” (including synonyms) was registered in the EHR in 2019.

Patients were excluded if (1) it was registered in the EHR that the patient granted no permission to use their data for scientific research; (2) DTC G17 (endometriosis), G18 (contraception), M14 (malignancy endometrium), 0313 741 (hemophilia), 0313 742 (von Willebrand disease), or 0313 749 (other coagulation disorders) were registered in 2019; and (3) the diagnosis codes or textual terms (including synonyms) “uterine polyp,” “cesarean scar niche,” “endometriosis (including adenomyosis),” “endometrial malignancy,” “postmenopausal vaginal bleeding,” or “coagulopathies” were registered in the EHR in 2019.

The CDC selected patients according to the in‐ and exclusion criteria. The combination of structured and unstructured data, together with the use of a CDC, allowed a standardized method to identify patients with fibroid‐related HMB. One researcher (LK) validated this selection and determined the final inclusion or exclusion based on the pseudonymized free‐text report extracted from the EHR.

### 2.4. Data Extraction and Validation

Data were extracted between August 2023 and December 2024. Data were collected from the date of the first consult in 2019 until the last possible visit in December 2022, so all patients had at least three years of follow‐up. Hospital‐specific queries were built in the CDC. The query was configured to extract the oldest result from the EHR. This way, the first treatment initiated after the primary consultation was extracted and stepped care could be identified. Both structured and unstructured data were retrieved. Structured variables included age, country of birth (Dutch or non‐Dutch), vital signs, diagnoses, DTCs, HCAC, and pharmaceuticals using Anatomic Therapeutic Chemical codes. Unstructured variables were the localization of (dominant) fibroid(s) (intracavitary, submucosal, intramural, subserosal, or transmural), number of fibroids (solitary, multiple, or multiple with a dominant fibroid), and presence of an intracavitary component. Regarding fibroid localization, both the FIGO classification and the terms intracavitary, submucosal, intramural, subserosal, and transmural were extracted. Due to underreporting, it was decided to combine the FIGO classification and terms, and report the fibroid localization based on terms.

One researcher (LK) validated the unstructured data using the pseudonymized free‐text report extracted from the EHR. Data were exported and transferred to an SPSS file after data cleansing.

Medication prescribed within 2 days of any other treatment than pharmacological treatment was not included as pharmacological treatment, because nonsteroidal anti‐inflammatory drugs (NSAIDs) are often prescribed as analgesics for these treatments. If a gonadotropin‐releasing hormone agonist (GnRHa) was prescribed 6 months prior to myomectomy or hysterectomy, this was considered surgical pretreatment and therefore not registered as pharmacological treatment. GnRHas, like relugolix and linzagolix, are available in the Netherlands since July 2021 and Juni 2022. Relugolix is reimbursed since December 2022, whereas linzagolix is not yet part of reimbursed care [18, 19]. Since data were extracted until December 2022 and these agents were not yet part of standard fibroid care in the Netherlands during this period, GnRHas were excluded from data extraction.

The HCAC 037177 includes both therapeutic hysteroscopy and endometrial ablation. When HCAC 037177 and an appointment type for endometrial ablation were registered on the same day, this was registered as an endometrial ablation.

### 2.5. Outcomes

The primary outcome was the median number of treatments that were initiated from the first outpatient consultation in 2019 throughout December 2022. Treatments were subdivided in pharmacological treatment (NSAIDs, tranexamic acid, combined oral contraceptive pill, progestogen‐only pill, contraceptive vaginal ring or patch, GnRHa, ulipristal acetate and hormonal intrauterine device [IUD]) and (minimally) invasive interventions (therapeutic hysteroscopy, endometrial ablation, radiofrequency ablation [RFA], UAE, magnetic resonance–guided high‐intensity focused ultrasound [MR‐HIFU], myomectomy, and hysterectomy).

Secondary outcomes were the type of treatment initiated, the number and type of treatment prior to hysterectomy, and the time to a hysterectomy from the first presentation in the outpatient clinic. Regarding hysterectomy, possible confounders and effect modifiers were tested in the association between hospital and undergoing hysterectomy.

### 2.6. Statistical Analysis

Statistical analysis was performed using SPSS Version 26 (IBM Corporation, Armonk, New York, USA). Data were presented stratified by hospital and in total. Missing data were reported. Continuous variables were presented as mean (standard deviation) when normally distributed and as median (range) when non‐normally distributed. Differences in continuous variables between the hospitals were tested using one‐way ANOVA tests for normally distributed data and Kruskal–Wallis tests for non‐normally distributed data. Categorical data were presented as *n* (%). Differences in categorical data between hospitals were assessed using chi‐square tests. A *p* value < 0.05 was considered statistically significant.

Kaplan–Meier survival analysis was conducted for time to hysterectomy from the first appointment. A logistic regression model was estimated, to test for potential confounders and effect modifiers in the association between hospital and undergoing hysterectomy (yes/no). Hospital 1 was taken as the reference hospital, because it had the highest number of included patients and hysterectomies performed. Age, BMI, ethnicity, intracavitary component, and the number of fibroids were analyzed as possible confounders or effect modifiers. When the regression coefficient changed ≥ 10%, the variable was considered a confounder [20]. When the *p* value of the interaction was < 0.05, the variable was considered an effect modifier.

### 2.7. Data Discussion

The data were presented and discussed in a semistructured online meeting with the researchers and one delegated gynecologist from each hospital. The participants received the data prior to the meeting. The delegated gynecologists were asked to discuss the results with their business managers prior to the meeting. The aim of this discussion was to identify possible (organizational) causes of CPV.

## 3. Results

### 3.1. Characteristics of Study Population

**FIGURE 1:**
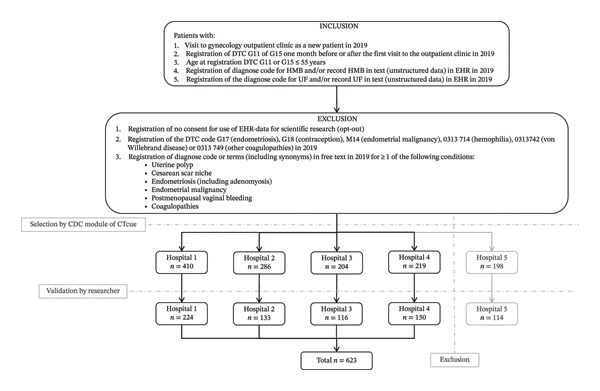
Study population flowchart including the inclusion and exclusion criteria. After the first selection by the Clinical Data Collector (CDC) based on the inclusion and exclusion criteria, the inclusion of all selected patients was validated by one researcher in the pseudonymized free‐text report extracted from the electronic health records (EHR) in the CDC. The researcher established final inclusion and exclusion based on the inclusion and exclusion criteria. The patients of Hospital 5 were excluded as it appeared that many healthcare activity codes (HCAC) were not correctly registered in Hospital 5. This resulted in the inclusion of 623 patients in total from four hospitals. CDC = Clinical Data Collector, DTC = diagnosis treatment combination, EHR = electronic health records.

After data extraction, it appeared that many HCAC were not correctly registered in Hospital 5. Therefore, data from this hospital were excluded from statistical analysis, as this would not provide an accurate representation of treatments provided and would affect the results. This resulted in the inclusion of 623 women from four hospitals (Figure 1). The median follow‐up time was 42 months (range: 36–47 months). The median age was 46 years (range: 23–55 years), and the median BMI 26.0 kg/m^2^ (range: 15.7–37.0 kg/m^2^). FIGO classification was not reported in 68.7% (428/623) patients. Fibroids had an intracavitary component in 196 patients (31.5%). Solitary fibroids were present in most patients (366/623 [58.7%]). There was no significant interhospital variation in the characteristics of the study population (Table 1).

**TABLE 1:** Patient characteristics.

	Hospital 1	Hospital 2	Hospital 3	Hospital 4	Total	*p* value
*N*	224 (100.0)	133 (100.0)	116 (100.0)	150 (100.0)	623 (100.0)	

Follow‐up time in months (median [range])	42 (36–47)	42 (36–47)	42 (36–47)	42 (36–47)	42 (36–47)	0.98

Non‐Dutch ethnicity (missing data)	36 (16.1)	34 (25.6)	19 (16.4)	28 (18.7)	117 (18.8)	0.14
	(*n = *57)	(*n = *99)	(*n = *50)	(*n = *42)	(*n = *248)	

Age (median [range])	46 (29–55)	47 (27–55)	47 (23–55)	46 (24–55)	46 (23–55)	0.20
(Missing data)	(*n = *1)	(*n = *0)	(*n = *0)	(*n = *7)	(*n = *8)	

BMI (median [range])	25.9 (18.2–52.7)	25.2 (16.9–41.9)	26.3 (17.9–41.0)	27.5 (15.7–43.3)	26.0 (15.7–37.0)	0.16
(Missing data)	(*n = *102)	(*n = *62)	(*n = *60)	(*n = *83)	(*n = *307)	

History of CS (missing data)	44 (19.6)	20 (15.0)	23 (19.8)	20 (13.3)	107 (17.2)	0.32
	(*n = *0)	(*n = *0)	(*n = *0)	(*n = *0)	(*n = *0)	

History of DVT/PE/infarction (missing data)	13 (5.8)	6 (4.5)	6 (5.2)	12 (8.0)	37 (5.9)	0.63
	(*n = *0)	(*n = *0)	(*n = *0)	(*n = *0)	(*n = *0)	

History of breast cancer (missing data)	0 (0.0)	1 (0.8)	1 (0.9)	2 (1.3)	4 (0.6)	0.44
	(*n = *0)	(*n = *0)	(*n = *0)	(*n = *0)	(*n = *0)	

Number of uterine fibroids						0.22
Solitary	134 (59.8)	70 (52.6)	76 (65.5)	86 (57.3)	366 (58.7)	
Multiple	72 (32.1)	53 (39.8)	39 (33.6)	55 (36.7)	219 (35.2)	
Multiple, dominant (missing data)	17 (7.6)	10 (7.5)	1 (0.9)	9 (6.0)	37 (5.9)	
	(*n = *1)	(*n = *0)	(*n = *0)	(*n = *0)	(*n = *1)	

Location of uterine fibroids						0.10
Submucosal (FIGO 0–2)	30 (13.4)	24 (18.0)	36 (31.0)	19 (12.7)	109 (17.5)	
Intramural (FIGO 3–4)	10 (4.5)	10 (7.5)	20 (17.2)	0 (0.0)	40 (6.4)	
Subserosal (FIGO 5–7)	4 (1.8)	2 (1.5)	15 (12.9)	3 (2.0)	24 (3.9)	
Cervical (FIGO 8)	0 (0.0)	0 (0.0)	3 (2.6)	0 (0.0)	3 (0.5)	
Transmural (FIGO 2–5)	0 (0.0)	1 (0.8)	8 (6.9)	0 (0.0)	9 (1.4)	
(Missing data)	(*n = *186)	(*n = *100)	(*n = *56)	(*n = *86)	(*n = *428)	

Intracavitary component (missing data)	73 (32.6)	34 (25.6)	40 (34.5)	49 (32.7)	196 (31.5)	0.38
	(*n = *18)	(*n = *8)	(*n = *5)	(*n = *12)	(*n = *43)	

DTC						0.35
G11	129 (57.6)	87 (65.4)	75 (64.7)	90 (60.0)	381 (61.2)	
G15 (missing data)	95 (42.4)	46 (34.6)	41 (35.3)	49 (32.7)	231 (37.1)	
	(*n = *0)	(*n = *0)	(*n = *0)	(*n = *12)	(*n = *12)	

*Note:* Presented as *n* (%), unless indicated otherwise.

Abbreviations: CS = cesarean section, DTC = diagnosis treatment combination, DVT = deep venous thrombosis, FIGO = International Federation of Gynecology and Obstetrics, PE = pulmonary embolism.

### 3.2. Primary Outcome: Number of Fibroid Treatments

The primary outcome, the number of fibroid treatments initiated per patient over ≤ 3 years, varied significantly across the four hospitals (Table 2, *p* < 0.01). All hospitals had a median of one treatment, but the range differed between Hospitals 1 and 3 (range: 1–4) compared to Hospitals 2 and 4 (range: 1–3).

**TABLE 2:** Prevalence of treatments received by included patients, stratified per hospital, and in total.

	Hospital 1 (*n = *224)	Hospital 2 (*n = *133)	Hospital 3 (*n = *116)	Hospital 4 (*n = *150)	Total (*n = *623)	*p* value
Number of treatments (median [range])	1 (1–4)	1 (1–3)	1 (1–4)	1 (1–3)	1 (1–4)	**< 0.01**

Any treatment	200 (89.3)	104 (78.2)	86 (74.1)	117 (78.0)	507 (81.4)	**< 0.01**
No treatments	24 (10.7)	29 (21.8)	30 (25.9)	33 (22.0)	116 (18.6)	**< 0.01**
One treatment	109 (48.7)	68 (51.5)	48 (41.4)	75 (50.0)	300 (48.2)	0.42
Two treatments	74 (33.0)	35 (26.3)	24 (20.7)	38 (25.3)	171 (27.4)	0.09
Three treatments	14 (6.3)	1 (0.8)	11 (9.5)	4 (2.7)	30 (4.8)	**< 0.01**
Four treatments	3 (1.3)	0 (0.0)	3 (2.6)	0 (0.0)	6 (1.0)	**< 0.01**

Any pharmacological treatment	158 (70.5)	86 (64.7)	57 (49.1)	91 (60.7)	392 (62.9)	**0.02**
NSAIDs	13 (8.2)	17 (19.8)	6 (10.5)	10 (11.0)	46 (11.7)	0.06
Tranexamic acid	95 (60.1)	31 (36.0)	29 (50.9)	51 (56.0)	206 (52.6)	**< 0.01**
Combined oral contraception pill	27 (17.1)	17 (19.8)	12 (21.1)	25 (27.5)	81 (20.7)	0.44
Progestin‐only pill	45 (28.5)	14 (16.3)	9 (15.8)	17 (18.7)	85 (21.7)	**< 0.01**
Contraceptive vaginal ring/patch	0 (0.0)	0 (0.0)	1 (1.8)	1 (1.1)	2 (0.5)	0.43
GnRHa	9 (5.7)	9 (10.5)	4 (7.0)	17 (18.7)	39 (9.9)	**0.02**
Ulipristal	2 (1.3)	2 (2.3)	5 (8.8)	7 (7.7)	16 (4.1)	0.07
Hormonal IUD	61 (38.6)	32 (37.2)	21 (36.8)	33 (36.3)	147 (37.5)	0.28

Any (minimally) invasive treatment	132 (58.9)	54 (40.6)	66 (56.9)	67 (44.7)	319 (51.2)	**< 0.01**
Therapeutic hysteroscopy	30 (22.7)	21 (38.9)	28 (42.4)	33 (49.3)	112 (35.1)	**< 0.01**
Endometrial ablation	39 (29.5)	16 (29.6)	17 (25.8)	21 (31.3)	93 (29.2)	0.91
RFA (research setting)	0 (0.0)	0 (0.0)	10 (15.2)	0 (0.0)	10 (3.1)	**< 0.01**
UAE	1 (0.8)	3 (5.6)	2 (3.0)	2 (3.0)	8 (2.5)	0.28
MR‐HIFU (research setting)	12 (9.1)	0 (0.0)	0 (0.0)	0 (0.0)	12 (3.8)	**< 0.01**
Myomectomy	2 (1.5)	1 (1.9)	3 (4.5)	0 (0.0)	6 (1.9)	0.27
Pretreatment with GnRHa	1 (50.0)	1 (100.0)	2 (66.7)	0 (0.0)	4 (66.7)	0.69
Abdominal myomectomy	2 (100.0)	0 (0.0)	3 (100.0)	0 (0.0)	5 (83.3)	**0.05** ^1^
Laparoscopic myomectomy	0 (0.0)	1 (100.0)	0 (0.0)	0 (0.0)	1 (16.7)	
Hysterectomy	69 (52.3)	14 (25.9)	24 (36.4)	16 (23.9)	123 (38.6)	**< 0.01**
Pretreatment with GnRHa	3 (4.3)	2 (14.3)	8 (33.3)	4 (25.0)	17 (13.8)	**0.02**
Abdominal hysterectomy	21 (30.4)	0 (0.0)	0 (0.0)	1 (6.3)	22 (17.9)	**0.04** ^2^
Laparoscopic hysterectomy	39 (56.5)	12 (85.7)	20 (83.3)	13 (81.3)	84 (68.3)	
Supravaginal hysterectomy	5 (7.2)	0 (0.0)	0 (0.0)	0 (0.0)	5 (4.1)	
Vaginal hysterectomy	4 (5.8)	2 (14.3)	4 (16.7)	2 (12.5)	12 (9.8)	

*Note:* Presented as *N* (%) unless mentioned otherwise. Bold *p* values are statistically significant, *p* < 0.05.

Abbreviations: GnRHa = gonadotropin‐releasing hormone agonist, IUD = intrauterine device, MR‐HIFU = magnetic resonance–guided high‐intensity focused ultrasound, NSAIDs = nonsteroidal anti‐inflammatory drugs, RFA = radiofrequency ablation, UAE = uterine artery embolization.

^1^Concerns the method of myomectomy.

^2^Concerns the method of hysterectomy.

### 3.3. Secondary Outcomes: Treatment Intensity, Prevalence, and Modality

Overall, 18.6% of patients (116/623) did not receive any treatment during the study period. The proportion of untreated patients varied between hospitals, ranging from 10.7% in Hospital 1 to 25.9% in Hospital 3 (*p* < 0.01). Conversely, 81.4% of patients (507/623) underwent at least one intervention. The proportion treated was highest in Hospital 1 (89.3%) and lowest in Hospital 3 (74.1%) (*p* < 0.01). Single‐modality therapy predominated (48.2% overall, peaking at 51.5% in Hospital 2). The use of two or more modalities was most common in Hospital 1 (40.6%) and was markedly lower in Hospitals 2 (27.1%), 3 (32.8%), and 4 (33.2%). Extensive management (four modalities) was rare, affecting 1.0% of patients (Table 2).

**FIGURE 2:**
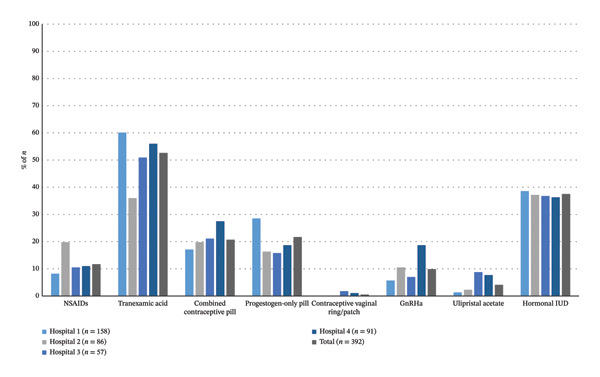
Visual representation of the prevalence of pharmacological treatments received by included patients, stratified per hospital, and in total. The number of pharmacological treatments is presented as the percentage of total number of patients that received pharmacological treatment per hospital or in total. IUD = intrauterine device, NSAIDs = nonsteroidal anti‐inflammatory drugs, GnRHa = gonadotropin‐releasing hormone agonist.

**FIGURE 3:**
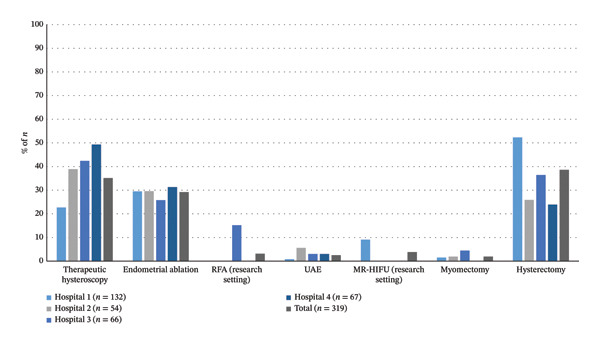
Visual representation of the prevalence of (minimally) invasive treatments received by included patients, stratified per hospital, and in total. The number of treatments is presented as the percentage of total number of patients that received any (minimally) invasive treatment per hospital or in total. MR‐HIFU = magnetic resonance–guided high‐intensity focused ultrasound, RFA = radiofrequency ablation, UAE = uterine artery embolization.

Pharmacological treatment was the most frequently initiated modality across all hospitals (overall 392/623 [62.9%], range: 49.1%–70.5%), although rates differed significantly (*p* = 0.02) (Table 2) (Figure 2). The most frequently used pharmacological agents were tranexamic acid (52.6%), progestin‐only pills (21.7%), and combined oral contraceptives (20.7%). NSAIDs were prescribed in 11.7%, and GnRHa in 9.9% of treated patients. Less common were ulipristal acetate (4.1%) and the vaginal ring/patch (0.5%). Notable interhospital differences were observed in the choice of tranexamic acid (*p* < 0.01), progestin‐only pills (*p* < 0.01), and GnRHa agents (*p* = 0.02). Hormonal IUD was used in 37.5% of patients, with no interhospital difference (*p* = 0.28). The specification of prescribed combined oral contraceptive pills, progestin‐only pills, and GnRHa is presented in Supporting Information Table S1.

(Minimally) invasive treatments were applied in 51.2% (319/623) of patients, ranging from 40.6% to 58.9% among hospitals (*p* < 0.01) (Table 2) (Figure 3). Therapeutic hysteroscopy was overall applied in 35.1% (112/319), but most often in Hospital 4 (49.3%) (*p* < 0.01). Of the patients receiving any (minimally) invasive treatment option, only 9.4% (30/319) received one of the uterus‐sparing options RFA (*n* = 10, *p* < 0.01), MR‐HIFU (*n* = 12, *p* < 0.01), or UAE (*n* = 8, *p* = 0.28). Myomectomy was performed in 1.9% of patients (6/319), which did not differ among hospitals (*p* = 0.27), but the surgical method did (*p* = 0.05).

Overall, hysterectomy was performed in 38.6% of the patients (123/319). Hospital 1 had the highest hysterectomy rate at 52.3% (69/132) (*p* < 0.01), compared to 23.9%–36.4% in the other hospitals, reflecting a significant interhospital variation in the use of definitive surgical management (Table 2) (Figure 3). The median time from the first consultation at the hospital to hysterectomy was 4 months. Kaplan–Meier survival analysis showed no significant differences in time to hysterectomy between hospitals (*p* = 1.00) (Table 3) (Supporting Information Figure S1). The use of GnRHa pretreatment and the surgical approach differed significantly between hospitals (*p* = 0.02 and *p* = 0.04, respectively). Hospital 1 performed the least laparoscopic hysterectomies (56.6%) compared to the other hospitals (range: 81.3%–85.7%) while performing most abdominal hysterectomies (30.4%) compared to the other hospitals (range: 0.0%–6.3%) (Table 2).

**TABLE 3:** Number of treatments and type of treatment before hysterectomy, stratified per hospital, and in total.

	Hospital 1 (*n = *69)	Hospital 2 (*n = *14)	Hospital 3 (*n = *42)	Hospital 4 (*n = *16)	Total (*n = *123)	*p* value
Number of treatments before hysterectomy (median [range])	1 (0–3)	0 (0–2)	1 (0–3)	1 (0–2)	1 (0–3)	0.29

Any treatment	47 (68.1)	6 (42.9)	13 (54.2)	9 (56.3)	75 (61.0)	0.50
No treatments before hysterectomy	22 (31.9)	8 (57.1)	11 (45.8)	7 (43.8)	48 (39.0)	0.26
One treatment before hysterectomy	33 (47.8)	5 (35.7)	6 (25.0)	7 (43.8)	51 (41.5)	0.26
Two treatments before hysterectomy	11 (15.9)	1 (7.1)	5 (20.8)	2 (12.5)	19 (15.4)	0.71
Three treatments before hysterectomy	3 (4.3)	0 (0.0)	2 (8.3)	0 (0.0)	5 (4.1)	0.49

Time to hysterectomy (months [median (range)])	4 (0–41)	4.5 (1–28)	4 (1–29)	4.5 (1–23)	4 (0–41)	1.00^1^

Pharmacological treatment	46 (66.7)	6 (42.9)	13 (31.0)	9 (56.3)	74 (60.2)	0.33
NSAIDs	4 (8.7)	1 (16.7)	3 (23.1)	0 (0.0)	8 (10.8)	0.32
Tranexamic acid	36 (78.3)	5 (83.3)	4 (30.8)	6 (66.7)	51 (68.9)	**0.01**
Combined oral contraception pill	7 (15.2)	0 (0.0)	4 (30.8)	1 (11.1)	12 (16.2)	0.34
Progestin‐only pill	20 (43.5)	0 (0.0)	1 (7.7)	0 (0.0)	21 (28.4)	**< 0.01**
Contraceptive vaginal ring/patch	0 (0.0)	0 (0.0)	1 (7.7)	0 (0.0)	1 (1.4)	0.19
GnRHa	1 (2.2)	1 (16.7)	0 (0.0)	2 (22.2)	4 (5.4)	**0.04**
Ulipristal	1 (2.2)	0 (0.0)	4 (30.8)	3 (33.3)	8 (10.8)	**< 0.01**
Hormonal IUD	11 (23.9)	2 (33.3)	4 (30.8)	3 (33.3)	20 (27.0)	0.89

Any (minimally) invasive therapy	15 (21.7)	1 (7.1)	7 (16.7)	2 (12.5)	25 (20.3)	0.34
Therapeutic hysteroscopy	8 (53.3)	0 (0.0)	3 (42.9)	1 (50.0)	12 (48.0)	0.76
Endometrial ablation	5 (33.3)	1 (100.0)	2 (28.6)	1 (50.0)	9 (36.0)	0.54
RFA (research setting)	0 (0.0)	(0.0)	3 (42.9)	0 (0.0)	3 (12.0)	**0.03**
UAE	0 (0.0)	(0.0)	1 (14.3)	0 (0.0)	1 (4.0)	0.44
MR‐HIFU (research setting)	5 (33.3)	(0.0)	0 (0.0)	0 (0.0)	5 (20.0)	0.24
Myomectomy	0 (0.0)	(0.0)	0 (0.0)	0 (0.0)	0 (0.0)	—

*Note:* Presented as *N* (%) unless mentioned otherwise. Bold values indicate statistically significance, *p* < 0.05.

Abbreviations: GnRHa = gonadotropin‐releasing hormone agonist, IUD = intrauterine device, MR‐HIFU = magnetic resonance–guided high‐intensity focused ultrasound, NSAIDs = nonsteroidal anti‐inflammatory drugs, RFA = radiofrequency ablation, UAE = uterine artery embolization.

^1^Results of Kaplan–Meier survival analysis.

Overall, 48/123 patients (39.0%) underwent hysterectomy as the primary treatment (Table 3). There were no interhospital differences in the number of treatments prior to hysterectomy (*p* = 0.29). The types of treatments prior to hysterectomy did not differ statistically between hospitals, except for the number of RFA procedures (*p* < 0.01). Despite no interhospital differences in the initiation of pharmacological treatment prior to hysterectomy (*p* = 0.50), the usage of tranexamic acid, progestin‐only pill, GnRHa, and ulipristal acetate did differ (*p* = 0.01, *p* < 0.01, *p* = 0.04, and *p* < 0.01, respectively). Nine hysterectomies (7.3%) were performed after minimally invasive therapies (RFA, MR‐HIFU or UAE).

No confounders or effect modifiers were found in the association between hospital and the likelihood of undergoing hysterectomy (data not shown).

### 3.4. Data Discussion

To further understand the factors contributing to the observed CPV, the data were discussed with the hospitals during a reflection meeting. Hospital‐specific organizational factors contributing to CPV were identified. At Hospital 3, a one‐stop outpatient clinical evaluation for patients with fibroids was offered, with a diagnostic and therapeutic hysteroscopy directly available. Hospital 1 conducted a randomized clinical trial investigating (cost‐)effectiveness of MR‐HIFU compared to standard care for symptomatic uterine fibroids during the study period [21]. Hospital 3 conducted a prospective cohort trial investigating (cost‐)effectiveness of RFA for symptomatic uterine fibroids during the study period. Even though UAE was continuously offered in Hospitals 1 and 2 during the study period, the representatives commented that there was no dedicated interventional radiologist present during the study period, possibly limiting the UAE procedures performed in said hospitals. No other hospital‐specific organizational factors contributing to CPV were identified.

## 4. Discussion

This multicenter retrospective study revealed substantial CPV in the treatment of fibroid‐related HMB across four Dutch hospitals. The number and type of treatment per patient, as well as the proportion of untreated patients, varied significantly. These differences occurred despite similar patient and fibroid characteristics, suggesting that the observed variation is at least partly unwarranted. Indeed, discussions with the hospital representatives revealed that organizational factors partly explained the observed CPV.

The median number of treatments initiated per patient was one (range: 1–4), which differed significantly among hospitals. The proportion of untreated patients also differed considerably, suggesting a variation in thresholds for initiating treatment or differences in patient counseling. Pharmacological treatment was initiated in 62.9% of patients, (minimally) invasive treatments in 51.2%, which statistically differed among hospitals. Only 9.4% of patients received a minimally invasive uterus‐sparing treatment (RFA, MR‐HIFU, or UAE). A significant interhospital variation was present in the use of definitive surgical management and surgical methods. Hysterectomy was performed as primary treatment in 39.0%, which did not differ among hospitals. The median time to hysterectomy from the first consultation was 4 months.

Pharmacological therapy for HMB is often started as first‐line treatment following the stepped‐care approach. In line, we found that pharmacological treatment was the most frequently used modality, although its use varied across hospitals. Notably, there were differences in the choice of agents, particularly tranexamic acid, progestin‐only pills, and GnRHa, highlighting heterogeneity in initial management strategies. The choice for a pharmacological agent is dependent on individual patient preferences and expert opinion, especially in the prescription of oral contraceptive pills and progestin‐only pills [5]. This may explain the interhospital differences we observed in the usage of type of pharmacological agent, with patient preferences potentially reflecting warranted CPV. Our study shows that pharmacological therapy is still the most frequently prescribed modality in hospital care (overall 62.9%). This indicates that a substantial proportion of pharmacological management currently delivered in hospital settings could be shifted to primary care. With the availability of a diagnostic outpatient ultrasound service and adequate training of primary care physicians in pharmacological management of fibroids, such a shift may reduce referrals to the outpatient gynecology departments and subsequently reduce healthcare costs with preservation of healthcare quality [22].

Inconsistent use of the FIGO classification may partly explain the observed CPV in therapeutic hysteroscopy. Consistent use of the FIGO classification will lead to more consistent management [23]. It may result in more appropriate use of less invasive surgeries. For example, hysterectomy is not primarily indicated in cases of FIGO Type 0, 1, and 2 fibroids, as hysteroscopic myomectomy can be sufficient, if necessary, with pretreatment involving GnRHa or ulipristal acetate for anemia correction and presurgical volume reduction [24]. Nevertheless, our results showed that the FIGO classification was not reported in 68.7% of patients. This emphasizes the need to encourage consistent use of the FIGO classification among physicians to reduce unwarranted CPV. Additionally, easy access to diagnostic hysteroscopy, for example, during a one‐stop outpatient clinical evaluation as provided by Hospital 3, will aid correct fibroid classification and reduce unwarranted CPV.

In recent decades, there has been an increase in minimally invasive uterus‐preserving treatment options for fibroids, such as UAE, MR‐HIFU, and RFA. While MR‐HIFU and RFA are still limited to research settings in the Netherlands, UAE has been reimbursed and incorporated in Dutch guidelines since 2013 [25]. Nevertheless, only 2.5% of patients underwent UAE in this study. Although no interhospital variation in UAE was observed, the low percentage of UAE is remarkable, particularly given that the included patient population with a median age of 46 years falls within the typical eligibility range for UAE. This age group is nearing menopause, which reduces the likelihood of reintervention [26]. Moreover, fertility‐preserving treatment is often no longer a priority in this age group, relevant because UAE for women with fibroids who wish to preserve fertility is still controversial [26, 27]. Therefore, the limited use of UAE might indicate unwarranted CPV in all hospitals due to limited adoption of an evidence‐based guideline or, as the data discussion showed, due to the absence of a dedicated interventional radiologist. However, as literature studies have shown that hysterectomy rates have hardly decreased since UAE was incorporated into clinical guidelines, this may also indicate that UAE is not a replacement for hysterectomy, but rather an addition to fibroid care for patients seeking nonsurgical treatment [15, 28]. Lastly, the relatively recent introduction of minimally invasive treatment options may also contribute to a reluctance to implement these innovations. This reluctance among gynecologists has been documented by de Bruijn et al. who identified insufficient information and doubts about effectiveness as barriers [29]. In contrast, patients express a strong preference for high quality and complete information, and noninvasive, uterus‐ and fertility‐preserving options when selecting fibroid treatment [28, 30]. These contrasting perspectives emphasize the necessity of improving guideline adherence to reduce unwarranted CPV.

Targeted education and the presence of a clearly defined local treatment protocol can address the lack of information about minimal invasive treatments and improve adherence to guidelines. This will help physicians to provide objective, evidence‐based counseling on all available treatment options. Counseling formats, which can easily be integrated into the EHR, can support physicians in managing fibroids consistently. Additionally, Dutch physicians can actively refer patients to a fibroid‐specific decision aid to access reliable information on fibroid management [31].

The overall myomectomy rate in this study was low (1.9%) with no interhospital variation. Myomectomy is generally the preferred treatment for women with symptomatic uterine fibroids who are trying to conceive and for women with large fibroids who wish to preserve their fertility and for whom shrinkage would be insufficient [11]. The low myomectomy rate in this study may be due to the referral of patients to tertiary centers specialized in robot‐assisted or laparoscopic myomectomies, the absence of a current or future fertility desire leading to patients to opt for hysterectomy, or eligibility for alternative treatments such as therapeutic hysteroscopy, RFA, or MR‐HIFU. However, this information was not available in our study. Gaining this information would help to determine whether the low myomectomy rate is warranted or can be explained.

This study also showed CPV in the performance of hysterectomy and surgical approaches, which is in line with previous research [15]. Hospitals 1 and 3 performed most hysterectomies (52.3% and 36.4%, respectively), in contrast to lower hysterectomy rates of 25.9% and 23.9% in Hospitals 2 and 4, respectively. A possible explanation for the higher hysterectomy rates in Hospitals 1 and 3 is their participation in a clinical trial investigating (cost‐)effectiveness of MR‐HIFU and RFA during the study period [21]. Hospital representatives of Hospitals 1 and 3 commented that patients referred for trial screening often had large and/or multiple fibroids that made them ineligible for inclusion, potentially leading to hysterectomy as an alternative treatment. Furthermore, our results showed that hysterectomy was offered as the primary treatment in 39.0% of patients, without interhospital variation. This suggests that a stepped‐care approach is not applied even though this is advised in (inter)national guidelines, possibly indicating unwarranted CPV [5, 10, 11]. This deviation from guidelines might be partly explained by the relatively high age of the patient population. Previous studies show that overall hysterectomy rates rise with increasing age [30, 32]. However, no data were retrieved regarding the wish for fertility or uterus preservation, which might be an explanation for warranted CPV between hospitals in hysterectomy rates. Additionally, this study included only patients that presented as new patients in 2019 and were followed from that point onward. These patients might have received (stepped‐care) treatments prior to this presentation in primary care, another hospital, or within a previously opened and closed DTC. This may have led to an underestimation of prior treatment. The absence of interhospital differences in time to hysterectomy suggests that once a surgical trajectory was chosen, the speed of treatment was consistent.

Addressing unwarranted CPV in surgical approaches may benefit from a thorough evaluation of practice patterns at both physician and institutional levels. For example, if surgeons or surgical teams from certain hospitals are more likely to use an abdominal method than a laparoscopic method, despite similar patient and fibroid characteristics, this variation should be reduced through targeted education.

Lastly, as our results highlight, fibroid care is inherently multifactorial, which may contribute to the prevalence of CPV. Therefore, we recommend that (surgical) treatment plans for patients with fibroids are discussed within a dedicated multidisciplinary team, including (minimally invasive surgical) gynecologists and (interventional) radiologists. The roles of attendees regarding the indication for and eligibility of treatments should be clearly defined [33]. Such multidisciplinary meetings can be readily organized in the current digital era using online platforms, enabling multicentric participation and input from tertiary centers or centers offering treatments that are not locally available. Such a multidisciplinary approach has been shown to improve patient education regarding fibroids management, thereby reducing CPV based on individual physician preferences. [34, 35].

A major strength of this study is the usage of real‐world data, combining structured data with unstructured free‐text entries from EHRs. This automated approach enabled precise and standardized identification of patients with fibroid‐related HMB and provides a comprehensive view of clinical practice. By specifically excluding other causes for HMB than fibroids, we provide a detailed view of clinical practice patterns in this specific patient population compared to other studies [15]. Another strength was the integration of qualitative insights through discussions of the data with representatives of the hospitals. This added a valuable clinical perspective and the opportunity to identify and address unwarranted organizational factors for CPV, an approach not commonly addressed in previous studies.

The study also has its limitations. The use of unstructured data may introduce some bias, as its extraction by the CDC is dependent on registration in the EHR by the healthcare professional. As our results show (e.g., BMI or FIGO classification (Table 1)), data are often underreported. Furthermore, the CDC query was built to extract the oldest recorded treatment result, so it remains unclear whether a patient has undergone the same treatment again. Additionally, due to the structure of the Dutch healthcare system, treatments initiated in primary care or other hospitals, or treatments initiated within a previously opened and closed DTC before the study period were not captured. Furthermore, NSAIDs usage may be underreported as these are available in pharmacies without a prescription, and prescription of NSAIDs for analgetic purposes can influence the results as well. Lastly, data on important clinical variables such as uterine size, patient preferences regarding uterus or fertility preservation, or baseline hemoglobin values and the presence of HMB‐related anemia and its management were not available, which restricts our ability to comprehensively evaluate the suitability of treatment decisions and the extent of unwarranted variation.

Further research into CPV in fibroid management is needed to address the limitations of this study and to better understand the drivers of CPV and to address the unwarranted causes appropriately. To adequately investigate CPV, correct and consistent registration in the EHR is crucial. Based on our findings and expert opinion, supplemented by the literature, the authors developed a template that outlines the minimum data elements required to assess and document women with symptomatic uterine fibroids (Supporting Information Table S2) [36]. As research on preferences among patients with fibroids is currently lacking, and such insights are crucial for understanding the underlying reasons for CPV, we also recommend creating a dashboard including real‐time data on basic patient characteristics such as age, BMI, FIGO classification, DTCs, and HCAC, stratified by primary and secondary care. Such a dashboard would provide real‐time audit and feedback regarding practice patterns for fibroid care and will enable gynecologists to critically assess CPV and trends in care practice patterns. This data‐driven insight in combination with guideline regulations will improve healthcare for women with HMB and fibroids.

## 5. Conclusions

This multicenter, retrospective, database study demonstrated that a CPV is present in the treatment of fibroid‐related HMB in the Netherlands. This variation is unlikely the result of patient or fibroid characteristics, but could partially be explained by organizational factors. Sharing data between hospitals can aid in identifying, explaining, and acting on (un)warranted practice variation between healthcare clinics. While some degree of CPV remains inevitable, the extent observed here should be limited by ameliorating guideline adherence, educational interventions, and patient counseling, to improve the quality, efficiency, and equity of care.

**Nomenclature**
BMI: Body mass index
CDC: Clinical Data Collector
CPV: Clinical practice variation
DTC: Diagnosis treatment combination
EHR: Electronic health records
FIGO: International Federation of Gynecology and Obstetrics
GnRHa: Gonadotropin‐releasing hormone agonist
HCAC: Healthcare activity codes
HMB: Heavy menstrual bleeding
IUD: Intrauterine device
MR‐HIFU: Magnetic resonance–guided high‐intensity focused ultrasound
NSAIDs: Nonsteroidal anti‐inflammatory drugs
RFA: Radiofrequency ablation
UAE: Uterine artery embolization

## Author Contributions

Elisabeth R. Knorren: data curation, formal analysis, investigation, project administration, validation, visualization, and writing–original draft. Daniëlle P. C. Huijs: resources and writing–review and editing. Ingrid M. Nijholt: formal analysis, methodology, and writing–review and editing. Jeroen R. Dijkstra, F. Paul H. L. J. Dijkhuizen, Jan Willem van der Steeg, and Tycho van der Meer: writing–review and editing. Martijn F. Boomsma: supervision and writing–review and editing. Peggy M. A. J. Geomini, Maarten D. H. Vink, Marlies Y. Bongers, and Jaklien C. Leemans: conceptualization, methodology, and writing–review and editing.

## Funding

No funding was provided for this project.

## Disclosure

The research was conducted as part of the Heavy Menstrual Bleeding collaboration within the mProve confederation.

## Ethics Statement

The Medical Ethics Committee (MEC) of the Máxima Medical Centre, the initiating hospital, evaluated the study protocol (MEC Number: N22.037) and deemed it not applicable to the Medical Research Involving Human Subjects Act on June 9, 2022.

## Conflicts of Interest

The authors declare no conflicts of interest.

## Supporting Information

Figure S1. Kaplan–Meier survival analysis curve for the time to hysterectomy in months after the first consultation in the gynecologic outpatient clinic for heavy menstrual bleeding complaints in patients with uterine fibroids. Only patients who underwent hysterectomy were included in the Kaplan–Meier survival analysis. The Kaplan–Meier survival analysis showed no differences in the time to hysterectomy between the hospitals (*p* = 1.00).

Table S1. Template for the assessment of women with symptomatic uterine fibroids.

## Supporting information


**Supporting Information** Additional supporting information can be found online in the Supporting Information section.

## Data Availability

The data that support the findings of this study are available from the corresponding author upon reasonable request.
