# Supplementary material for: Treatment of Uterine Fibroid–Related Heavy Menstrual Bleeding: Variations in Clinical Practice at Four Hospitals in the Netherlands
Source: Obstet Gynecol Int. 2026 Feb 25;2026:2889686. doi: 10.1155/ogi/2889686 (PMC12936386; doi:10.1155/ogi/2889686)
Supplement: Supplementary file 1 — Supporting Information Additional supporting information can be found online in the Supporting Information section. [file OGI-2026-2889686-s001.zip › Supporting Information - Table S1.docx]

| Table S1 – Specification of combined oral contraceptive pills, progestin-only pills and GnRHa, stratified per hospital and in total | | | | | |
| --- | --- | --- | --- | --- | --- |
|  | **Hospital 1**  **(*n=*224)** | **Hospital 2**  **(*n=*133)** | **Hospital 3**  **(*n=*116)** | **Hospital 4**  **(*n=*150)** | **Total**  **(*n=*623)** |
| Any pharmacological treatment | 158 (70.5) | 86 (64.7) | 57 (49.1) | 91 (60.7) | 392 (62.9) |
| Combined oral contraception pill  Ethinylestradiol/levonorgestrel 30/150µg  Ethinylestradiol/levonorgestrel 20/100µg  Ethinylestradiol/levonorgestrel 50/125µg  Ethinylestradiol/drosperinon 30µg/3mg  Ethinylestradiol/drosperinon 20µg/3mg  Estradiol/nomegestrol 1.5/2.5mg  Estradiol/dienogest | 27 (17.1)  17 (63.0)  3 (11.1)  1 (3.7)  2 (7.4)  2 (7.4)  1 (3.7)  1 (3.7) | 17 (19.8)  14 (82.4)  1 (5.9)  0 (0.0)  0 (0.0)  1 (5.9)  0 (0.0)  1 (5.9) | 12 (21.1)  11 (91.7)  0 (0.0)  1 (8.3)  0 (0.0)  0 (0.0)  0 (0.0)  0 (0.0) | 25 (27.5)  20 (80.0)  3 (12.0)  2 (8.0)  0 (0.0)  0 (0.0)  0 (0.0)  0 (0.0) | 81 (20.7)  62 (76.5)  7 (8.6)  4 (4.9)  2 (2.5)  3 (3.7)  1 (1.2)  2 (2.5) |
| Progestin-only pills  Medroxyprogesteron 10mg  Medroxyprogesteron 5mg  Lynestrenol 5mg  Desogestrel 0.075mg  Norethisteron 5mg  Etonogestrel 68mg | 45 (28.5)  26 (57.8)  2 (4.4)  12 (26.7)  5 (11.1)  0 (0.0)  0 (0.0) | 14 (16.3)  1 (7.1)  0 (0.0)  9 (64.3)  3 (21.4)  1 (7.1)  0 (0.0) | 9 (15.8)  6 (66.7)  1 (11.1)  0 (0.0)  1 (11.1)  1 (11.1)  0 (0.0) | 17 (18.7)  4 (23.5)  0 (0.0)  8 (47.1)  1 (5.9)  3 (17.6)  1 (5.9) | 85 (21.7)  37 (43.5)  3 (3.5)  29 (34.1)  10 (11.8)  5 (5.9)  1 (1.2) |
| GnRHa  Leuproreline 3.75mg  Leuproreline 5mg  Leuproreline 11.25mg  Gosereline 3.6mg  Triptoreline 0.5mg/ml  Triptoreline 0.1mg/ml | 9 (5.7)  6 (66.7)  0 (0.0)  3 (33.3)  0 (0.0)  0 (0.0)  0 (0.0) | 9 (10.5)  5 (55.6)  1 (11.1)  2 (22.2)  1 (11.1)  0 (0.0)  0 (0.0) | 4 (7.0)  2 (50.0)  0 (0.0)  1 (25.0)  1 (25.0)  0 (0.0)  0 (0.0) | 17 (18.7)  6 (35.3)  0 (0.0)  7 (41.2)  2 (11.8)  1 (5.9)  1 (5.9) | 39 (9.9)  19 (48.7)  1 (2.6)  13 (33.3)  4 (10.3)  1 (2.6)  1 (2.6) |
| Presented as N (%).  GnRHa = gonadotropin releasing hormone agonist, NSAIDs = non-steroidal anti-inflammatory drugs | | | | | |
