# Supplementary material for: Treatment of Uterine Fibroid–Related Heavy Menstrual Bleeding: Variations in Clinical Practice at Four Hospitals in the Netherlands
Source: Obstet Gynecol Int. 2026 Feb 25;2026:2889686. doi: 10.1155/ogi/2889686 (PMC12936386; doi:10.1155/ogi/2889686)
Supplement: Supplementary file 1 — Supporting Information Additional supporting information can be found online in the Supporting Information section. [file OGI-2026-2889686-s001.zip › Supporting Information - Table S2.docx]

| **Table S2. Template for assessment of women with symptomatic uterine fibroids** | |
| --- | --- |
| **Patient information** | |
| Name |  |
| Date of birth |  |
| Age |  |
| BMI |  |
| History of thrombo-embolic or cardiovascular event | □ No  □ Yes (specify below) |
| History of breast cancer | □ No  □ Yes (specify below) |
| Ethnicity |  |
| Type of complaints | □ Heavy menstrual bleeding  □ Bulk/mechanical  □ Sub- or infertility  □ Others (specify below) |
| Duration of complaints |  |
| Current hemoglobin level | mmol/L |
| Future childbearing wish | □ Yes  □ No  □ Uncertain |
| Therapies started in primary care | □ Pharmacological therapy (specify below)  □ Intra-uterine device (IUD) placement (specify type of IUD below) |
| Specific preferences concerning treatment | □ None  □ No hormonal treatment  □ Non-surgical  □ Fertility preserving  □ Uterus preserving |
| Other relevant patient history |  |
| **Evaluation of uterine fibroid on (transvaginal) ultrasound** | |
| Number of uterine fibroids | □ 1 □ 2 □ 3 □4 □5 □ >5 |
| Number of uterine fibroids | □ Solitary  □ Multiple fibroids without a dominant fibroid (specify number of fibroids below):  □ Multiple fibroids with a dominant fibroid (specify number of fibroids below): |
| Localization of uterine fibroid | □ Anterior uterine wall  □ Posterior uterine wall  □ Fundal  □ Others (specify below) |
| FIGO classification | □ 1 □ 2 □ 3 □ 4 □ 5 □ 6 □ 7 □ 8 □ 2-5 □ Others (specify below) |
| Maximum diameter of (dominant) uterine fibroid (mm) |  |
| Vascularization of uterine fibroid (power Doppler ultrasound) | Overall vessel pattern (in whole uterus):  □ Uniform  □ Non-uniform  Flow at the site of the capsule of the uterine fibroid:  □ No flow  □ Minimal flow  □ Moderate flow  □ Abundant flow |

| **Submucous** | 0 | Pedunculated intracavitary |
| --- | --- | --- |
|  | 1 | <50% intramural |
|  | 2 | ≥50% intramural |
| **Intramural** | 3 | Contracts endometrium, 100% intramural |
|  | 4 | 100% intramural |
| **Subserosal** | 5 | Subserosal, ≥50% intramural |
|  | 6 | Subserosal, <50% intramural |
|  | 7 | Subserosal pedunculated |
|  | 8 | Others (eg cervical, parasitic) |
| **Transmural** | 2-5 | Submucous and subserosal |
| **Hybrid** |  | Two numbers are listed separated by a hyphen. By convention the first refers to the relation with the endometrium while the second refers to the relationship to the serosa (eg FIGO 2-5, transmural). |


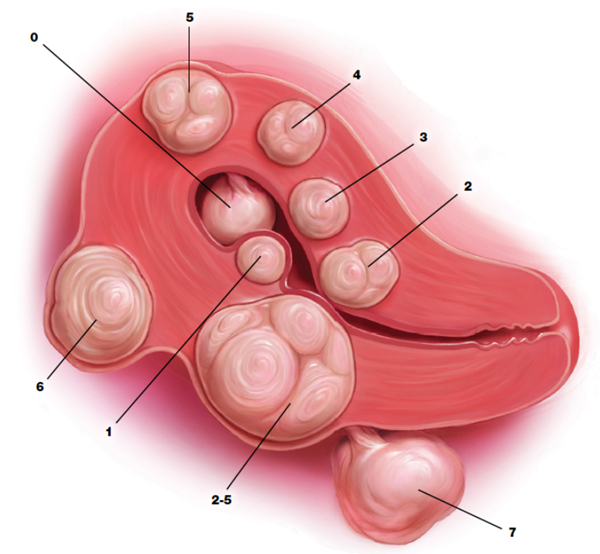
**FIGO classification**
