# Supplementary figures and images for: Treatment of Uterine Fibroid–Related Heavy Menstrual Bleeding: Variations in Clinical Practice at Four Hospitals in the Netherlands
Source: Obstet Gynecol Int. 2026 Feb 25;2026:2889686. doi: 10.1155/ogi/2889686 (PMC12936386; doi:10.1155/ogi/2889686)

## Slide 1
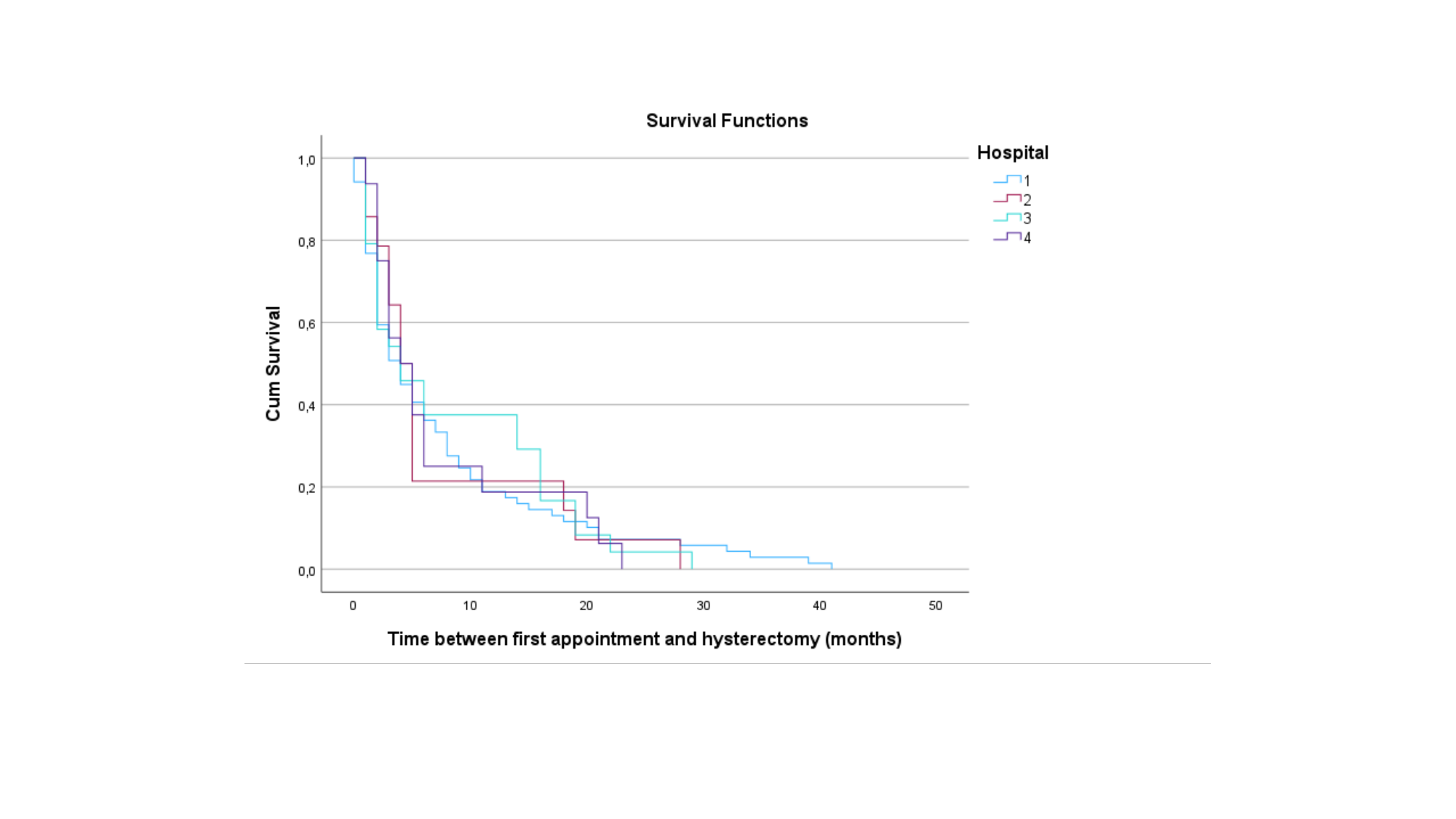

Supplement: Supplementary file 1 — Supporting Information Additional supporting information can be found online in the Supporting Information section. [file OGI-2026-2889686-s001.zip › Supporting Information - Figure S1 - Editable format.pptx]
